# Clinical and genetic features of congenital bile acid synthesis defect with a novel mutation in AKR1D1 gene sequencing: Case reports

**DOI:** 10.1097/MD.0000000000029476

**Published:** 2022-06-24

**Authors:** Anh-Hoa Nguyen Pham, Kim-Oanh Bui Thi, Mai-Huong Nguyen Thi, Diem-Ngoc Ngo, Nakayuki Naritaka, Hiroshi Nittono, Hisamitsu Hayashi, Trang Thi Dao, Kim-Huong Thi Nguyen, Hoai-Nghia Nguyen, Hoa Giang, Hung-Sang Tang, Tat-Thanh Nguyen, Dinh-Kiet Truong, Minh-Dien Tran

**Affiliations:** a Hepatology Department, National Children's Hospital, Hanoi, Vietnam; b Human Genetics Department, National Children's Hospital, Hanoi, Vietnam; c Junshin Clinic Bile Acid Institute, Tokyo, Japan; d Laboratory of Molecular Pharmacokinetics, Graduate School of Pharmaceutical Sciences, University of Tokyo, Tokyo, Japan; e Gene Solutions, Ho Chi Minh City, Vietnam; f Medical Genetics Institutes, Ho Chi Minh City, Vietnam; g University of Medicine and Pharmacy at Ho Chi Minh City, Vietnam.

**Keywords:** congenital bile acid synthesis defect, primary Δ4-3-oxosteroid 5β-reductase

## Abstract

**Rationale:**

Congenital bile acid synthesis defect (BASD) is a rare disease caused by mutations in the aldo-keto reductase 1D1 gene, which encodes the primary Δ4-3-oxosteroid 5β-reductase enzyme. Early disease diagnosis is critical for early treatment with bile acid replacement therapy, with an excellent chance for recovery. In contrast, protracted diagnosis and treatment may lead to poor outcomes, including decompensated hepatic cirrhosis, liver transplant, and even death.

**Patient concerns:**

Three clinical congenital bile acid synthesis defect cases in the Vietnamese population are herein reported. These pediatric patients presented with symptoms of prolonged postpartum jaundice and abnormal loose stool (mucus, lipids, and white). The clinical examinations showed hepatosplenomegaly. Urinalysis showed a very low fraction of primary bile acids and atypical 3-oxo-Δ4- bile acids in all three patients.

**Diagnoses:**

The patients were diagnosed with primary Δ4-3-oxosteroid 5β-reductase deficiency. Next-generation gene sequencing revealed two homozygous mutations in the aldo-keto reductase family 1 member D1 gene. The first is a documented variant, c.797G>A (p.Arg266Gln), and the second is a novel mutation at c.155T>C (p.Ile52Thr).

**Interventions:**

Immediately after diagnosis, patients were treated with oral chenodeoxycholate 5 mg/kg/d.

**Outcomes:**

The patients’ symptoms, signs, and primary bile acids levels improved significantly.

**Lessons:**

Clinicians should consider genetic disorders related to cholestasis for effective and life-saving treatment. A prompt genetic analysis by next-generation gene sequencing enables patients to access bile acid replacement therapy earlier, significantly improving short- and long-term outcomes.

## 1 Introduction

Congenital bile acid synthesis defect (BASD) type 2 is a rare autosomal recessive inherited disease caused by a defect in the aldo-keto reductase family 1 member D1 (*AKR1D1*) gene. This gene encodes Δ4-3oxosteroid 5β-reductase, which is primarily involved in bile acid biosynthesis.^[1,2]^ This disease was first described in case reports by Clayton et al and Setchell et al in 1988. ^[3,4]^ Clinically, patients with impaired bile acid synthesis present with intrahepatic cholestasis as early as a couple of weeks after delivery.^[5–7]^ The primary disease manifestations include progressive cholestatic jaundice, pruritis, white-colored stool, dark brown urine, and hepatosplenomegaly. Delay in diagnosis and treatment always leads to poor outcomes, including uncompensated liver cirrhosis, liver transplant, and death.

It is challenging to differentiate cholestasis caused by the primary 5β-reductase deficiency from other secondary etiologies in clinical practice. Therefore, genetic screening for *AKR1D1* mutations in clinically suspected patients is crucial for making an accurate diagnosis and early intervention.^[1–9]^ Furthermore, the replacement therapies by various types of cholic acids have been reported to effectively maintain liver function and prevent liver cirrhosis and the need for transplantation.^[5–7,10]^ Here, we report three cases of congenital congenital bile acid synthesis defect type 2 due to mutations in *AKR1D1*. Genetic analyses conducted at the National Pediatric Hospital in Hanoi, Vietnam, have unveiled a novel *AKR1D1* mutation. This report is expected to expand our sparse knowledge regarding this rare disease.

## 2 Case reports

### 2.1 Patient 1

**Figure 1:**
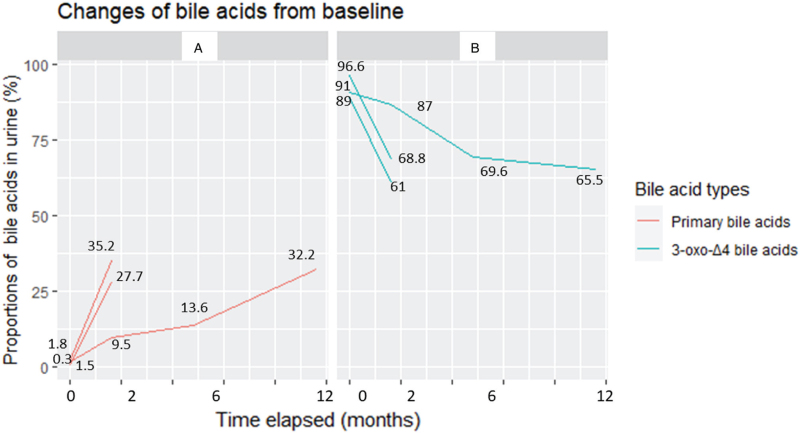
Dynamic variations in bile acid proportions (%) in urine samples from baseline to 12 months after bile acid replacement therapy. Figure 1A shows the increasing trend in primary bile acids, while Figure 1B presents the declining trend in 3-oxo-Δ4 bile acids in three patients.

**Figure 2:**
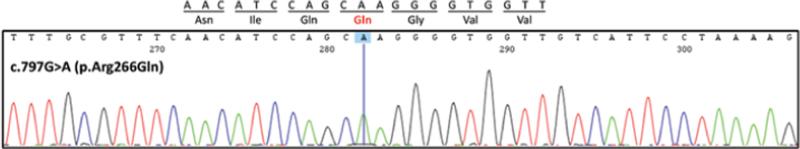
A homozygous mutation (G-to-A substitution) was found at the nucleotide 797 in the *AKR1D1* gene on chromosome 7, converting arginine into glutamine at position 266.

The 14-month-old male, from a full-term vaginal delivery, was his mother's third pregnancy. His parents were healthy and not consanguineous. Both his siblings were born healthy. Postpartum, the patient soon presented with prolonged jaundice and yellowish loose stool containing mucus and fats. On admission, the clinical examination showed substantial yellowing of the skin and eyes and hepatosplenomegaly; however, neither edema nor petechiae were observed. In addition, his condition revealed neither dysmorphic features nor a cardiac murmur. Laboratory tests showed a normal full blood count; transaminitis [aspartate aminotransferase (AST), 172 U/L and alanine aminotransferase (ALT), 108 U/L]; gamma-glutamyl transferase (GGT), 34 U/L; total bilirubin, 188 μmol/L, and direct bilirubin, 91 μmol/L; serum protein, 40 g/L; serum albumin, 26 g/L. While the total serum bile acid (TSBA) was 60 μmol/L, the proportion of primary bile acids in the urine was relatively low at 1.5%, contrasting with a 91% in atypical 3-oxo-Δ4 bile acid value at hospitalization (baseline), as illustrated in Figure 1. The blood lactate level was slightly elevated at 4.2 mmol/L. Coagulation tests showed mild dysfunction, with prothrombin time (PT) of 55% and an international normalized ratio (INR) of 1.52. Neither Epstein-Barr Virus (EBV) nor Cytomegalovirus (CMV) was detected by polymerase chain reaction (PCR) assay. Next-generation gene sequencing (NGs) test revealed a homozygous mutation in the *AKR1D1* gene at position c.797G>A (p.Arg266Gln), which converted arginine into glutamine (Fig. 2). The patient was diagnosed with primary Δ4-3-oxosteroid 5β-reductase deficiency. Afterward, gene sequencing of the patient's parents revealed that both are carriers of the heterozygous mutation c.797G>A in the *AKR1D1* gene. After confirmed diagnosis, the patient immediately started treatment with oral chenodeoxycholic acid (5 mg/kg/d). This therapeutic regimen was well tolerated. The patient was clinically stable with improvements in symptoms and signs. After 6 months of treatment, all laboratory tests were within normal ranges (Table 1).

**Table 1:** Clinical features, genetic analyses, management, and outcomes of three patients with congenital bile acid synthesis defect in Vietnam.

Case	Age and sex	Age at onset	Clinical features	Laboratory values on admission	PCR assays	Liver pathology	Treatment and outcomes	Genetic mutations
1	14-month-old male	First week after delivery	Prolonged jaundice, loose stool with mucus and lipids, hepatosplenomegaly, no dysmorphic feature	AST = 172 U/LALT = 108 U/LGGT = 34 U/LTotal bilirubin = 188 μmol/LDirect bilirubin = 91 μmol/LAlbumin = 26 g/LTotal bile acid = 60 mmol/LPT = 55%, INR = 1.52Lactate = 4.2 mmol/L	EBV, negativeCMV, negative	NA	Chenodeoxycholate 5 mg/kg/d, started soon after diagnosis. Complete clinical resolution and normal laboratory values after 6 mo of oral bile acid replacement therapy	*AKR1D1*, NM_005989.4: c.797G>A (p.Arg266Gln), homozygous
2	2-month-old male	First week after delivery	Prolonged jaundice, white loose stool, dark brown-colored urine, hepatosplenomegaly, lymphocele in the left thigh	AST = 442 U/LALT = 386 U/LGGT = 53 U/LTotal bilirubin = 145 μmol/LDirect bilirubin = 72 μmol/LAlbumin = 36 g/LTotal bile acid = 37 mmol/LPT = 71%, INR = 1.25Lactate = 2.8 mmol/L	EBV, negativeCMV, 6090 copies/mL	Giant multi-nuclear cell hepatitis, no fibrosis	Chenodeoxycholate 5 mg/kg/dWell treatment responseClinically stable and on recovery	*AKR1D1*, NM_005989.4: c.797G>A (p.Arg266Gln), homozygous
3	5-month-old male	First week after delivery	Prolonged jaundice, white loose stool, hepatosplenomegaly, no dysmorphic feature	AST = 982 U/LALT = 824 U/LGGT = 22 U/LTotal bilirubin = 245 μmol/LDirect bilirubin = 158 μmol/LTotal bile acid = 2.77 mmol/LPT = 41%, INR = 1.71	EBV, negativeCMV, negative	NA	Ursodeoxycholate 20 mg/kg/d started at five months old, limited treatment responseChenodeoxycholate 5 mg/kg/d was further indicated. He also continued Ursodeoxycholate. The patient showed partial response to therapeutic regimens.	*AKR1D1*, NM_005989.4: c.155T>C (p.Ile52Thr), homozygous

*AKR1D1* = aldo-keto reductase family 1 member D1 gene, ALT = alanine transferase (normal, <40), AST = aspartate aminotransferase (normal, <37), CMV = cytomegalovirus, EBV = Epstein-Barr virus, GGT = gamma-glutamyl transferase (normal, <45), INR = international normalized ratio (normal, <1.2), NA = not applicable, PCR = polymerase chain reaction, PT = prothrombin time (normal range from 85% to 100%).

### 2.2 Patient 2

This 2-month-old male patient, from a full-term vaginal delivery, was his mother's single offspring. The parents were healthy and not consanguineous. This patient presented with prolonged jaundice postpartum, accompanied by yellow switching to white-colored loose stool and dark, brown-colored urine. On admission, the physical examination revealed substantial jaundice in skin and eyes, mild hepatomegaly, unpalpable spleen, and neither edema nor petechiae. Remarkably, he had a small lymphocele located on his left thigh. Laboratory results showed mild anemia with a hemoglobin level of 105 g/L; total white blood cell count of 8120 cells/μl; normal platelet cell count, substantially elevated transaminases (ALT, 442 U/L and ALT, 386 U/L); total bilirubin, 145 μmol/L and direct bilirubin, 72 μmol/L; GGT, 53 U/L; serum protein, 54 g/L and albumin, 36 g/L. The serum TSBA was 37 μmol/L, and the percentage of primary bile acids in the urine was low at 1.8% compared with the high proportion of atypical 3-oxo-Δ4 bile acid at 89% at baseline (Fig. 1). The blood lactate was 2.8 mmol/L. The coagulation function tests were close to normal, with a PT of 71% and an INR of 1.25. As measured by quantitative PCR, the CMV load was 6090 copies/ml, whereas EBV was not detected. The magnetic resonance cholangiography showed normal findings with no evidence of congenital biliary atresia. The liver biopsy revealed images of giant multi-nuclear cell hepatitis and no fibrosis. NGs revealed a homozygous mutation in the *AKR1D1* gene, positioned c.797G>A, transforming arginine into glutamine (Fig. 2). The patient's mother also carried the heterozygous mutation c.797G>A in the *AKR1D1* gene. Primary Δ4-3-oxosteroid 5β-reductase deficiency was diagnosed. The child was treated with oral chenodeoxycholate 5 mg/kg/d immediately after confirmed diagnosis by the genetic analysis. This therapeutic regimen was well tolerated, and the patient became clinically stable and is in recovery (Table 1).

### 2.3 Patient 3

**Figure 3:**
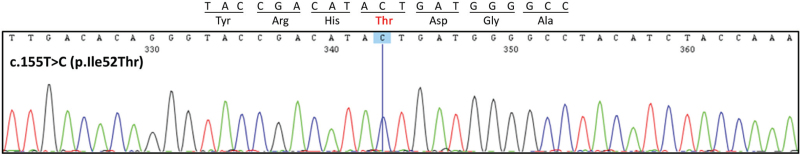
A homozygous mutation (T-to-C substitution) was identified at nucleotide 155 in the *AKR1D1* gene on chromosome 7, transforming isoleucine into threonine at position 52.

This 5-month-old male patient was a preterm (33-week gestational age) vaginal delivery from the south of Hanoi, Vietnam. The newborn had a birth weight of 1700 g and was his mother's single offspring. The parents were healthy and not consanguineous. This patient presented with prolonged jaundice soon after delivery, accompanied by yellow switching to white-colored loose stool. On admission, the physical examination revealed substantial jaundice of skin and eyes, hepatosplenomegaly, and neither edema nor petechiae. In addition, he showed neither dysmorphic features nor cardiac murmur. Laboratory results showed normal full blood counts, dramatically elevated transaminases (aspartate aminotransferase, 982 U/L and ALT, 824 U/L); GGT, 22 U/L; total bilirubin, 245 μmol/L and direct bilirubin, 158 μmol/L. On admission, the serum TSBA was 2.77 μmol/L, and the proportion of the primary bile acids was low at 0.3%, contrasted by the high percentage of atypical 3-oxo-Δ4 bile acid at 96.6% (Fig. 1). The blood lactate was slightly increased. The patient displayed a dysfunction in coagulation tests with a PT of 41% and an INR of 1.71. PCR assays detected neither EBV nor CMV. The genetic analysis of this patient revealed a homozygous mutation in the *AKR1D1* gene, positioned c.155T>C (p.Ile52Thr), transforming isoleucine into threonine at position 52 (Fig. 3). Gene sequencing of the patient's parents showed that both father and mother harbored an identical heterozygous mutation c.155T>C. The diagnosis of primary Δ4-3-oxosteroid 5β-reductase deficiency was established. The child was treated with oral ursodeoxycholic acid (UDCA) 20 mg/kg/d at 5 months old soon after diagnosis was confirmed by genetic analysis. However, he showed slight clinical and hepatic function improvements. Consequently, he was prescribed oral chenodeoxycholate 5 mg/kg/d while he continued UDCA treatment. The patient showed partial treatment response and is being followed up (Table 1).

### 2.4 Analysis of urine bile acids profile with mass spectrometry

Liquid chromatography-electrospray ionization tandem mass spectrometry (LC-ESI-MS/MS) was used to analyze various bile acids in urine samples as potential biomarkers to diagnose AKR1D1 deficiency and to monitor treatment response.^[15]^ Urine samples were collected from patients 1 and 2 during UDCA therapy (20 mg/kg/24 h). In patient 3, a urine sample was taken at age 5 months immediately upon diagnosis, without UDCA administration. The LC-ESI-MS/MS was used to perform quantitative analysis for different types of bile acids in the patients’ urine samples.^[15,16]^ On admission (baseline), all patients showed very low percentages of primary bile acids, including conjugated and unconjugated cholic acid and chenodeoxycholic acid of 1.5%, 1.8%, and 0.3%, respectively (Fig. 1). In contrast, significantly high proportions of atypical 3-oxo-Δ4- bile acids (including glycine and taurine conjugated 7α-hydroxy-3-oxo-Δ4-cholenoic acids and 7α, 12α-dihydroxy-3-oxo-Δ4-cholenoic acids) were observed at 91%, 89%, and 96.6%, respectively, corresponding to chenodeoxycholic acid replacement therapy over time. Dramatic increases in primary bile acids in parallel with substantial decreases in atypical 3-oxo-Δ4- bile acids were seen in these patients (Fig. 1A and 1B). These levels are typical of chenodeoxycholic acid replacement therapy.

### 2.5 Identification of *AKR1D1* gene defects

Both patient 1 and patient 2 presented identical homozygous mutations in the *AKR1D1* gene, in which there was a G-to-A substitution at nucleotide 797 (Fig. 2). A novel homozygous *AKR1D1* mutation located at nucleotide 155, causing a T-to-C substitution, was identified in patient 3 (Fig. 3).

## 3 Discussion

These cases demonstrate several important features of inborn errors of bile acid metabolism (IEBAM). Firstly, diagnosing infants with IEBAM poses a challenge in terms of non-specific signs and symptoms, which are indistinguishable from neonatal sepsis and various systemic diseases.^[3]^ In this study, the patients with IEBAM presented with apparent cholestatic jaundice and hepatosplenomegaly, occurring soon after delivery. In addition, reduced 5-β-reductase activity can be caused by several hepatotropic infections and metabolic disorders, further masking and complicating the diagnosis, as seen in patient 2, who had a considerable CMV load (Table 1).^[3]^ Secondly, because of the rarity of the IEBAM, comprising roughly 2% of neonatal cholestasis, a strong clinical suspicion is necessary for early diagnosis and management to improve patients’ outcomes; the disease is treated very effectively with bile acid replacement.^[5–7,10,12–14]^ We used the LC-ESI-MS/MS analysis to examine bile acids from dried urine sampling.^[15,16]^ The deficiency of Δ4-3-oxosteroid-5β-reductase causes defective bile acid steroid nucleus synthesis. The enzyme Δ4-3-oxosteroid-5β-reductase is encoded by the *AKR1D1* gene and converts 7α-hydroxy-4-cholesten 3-one and 7α,12α-dihydroxy-4-cholesten-3 one into 3-oxo-5β analogs.^[1,2]^ As a result, low levels of normal primary bile acids were present in the serum and urine of affected patients, while intermediate products of bile acid synthesis accumulated and could be detected by the LC-ESI-MS/MS. Therefore, the dynamic changes in proportions of the primary bile acids and 3-oxo-Δ4 bile acids were considered potential biomarkers to make a preliminary diagnosis and indicate a need for further genetic analysis to confirm (Fig. 1).^[16]^ Thirdly, our cases highlight the practical need for early disease detection by Sanger gene sequencing, to date regarded as the gold standard diagnostic tool. An NGs panel has been proposed as the first-line genetic diagnostic tool for IEBAM owing to its high throughput, accuracy, and reduced cost, given the genetic heterogeneity of IEBAM.^[17]^

The c.797G>A (p.Arg266Gln) homozygous mutation in the *AKR1D1* gene found in the first two patients in this study has been reported in several studies.^[7,18]^ However, the homozygous mutation c.155T>C (p.Ile52Thr) in the *AKR1D1* gene identified in patient 3 was a thoroughly novel variant.^[19]^ Thus, for the first time, we present this rare disease among the Vietnamese population and describe a novel mutation c.155 T>C, Ile to Thr substitution, which we added to the archive of *AKR1D1* variants. Lastly, to date, there has been no specific therapy for primary Δ4-3-oxosteroid 5β-reductase, and the mainstay therapy is bile acid replacement therapy. After genetic analyses confirmed *AKR1D1* deficiency, all patients were treated with chenodeoxycholic acid 5 mg/kg/d (Table 1). Patients 1 and 2 showed significant clinical improvement and normalized liver functions on oral bile acid replacement therapy, whereas patient 3 had a partial treatment response. Likewise, the significant biomarker for monitoring the patients’ treatment responses is the dynamic variations in the percentage of primary bile acids versus specific 3-oxo-Δ4 bile acids from baseline to post-treatment time points (Fig. 1). More specifically, the increasing trends in the primary bile acids in parallel with the declining trends in the specific 3-oxo-Δ4 bile acids may predict therapy response outcomes as observed in these three patients.

In conclusion, our cases demonstrate how NGs unmasked this rare bile acid metabolic disorder. This report urgently argues for protocol development, particularly for using LC-ESI-MS-MS as a screening tool for NGs among infants with prolonged neonatal cholestasis to detect this disease early and interrupt disease progression with preemptive cholic acid replacement therapies.

### 3.1 Uncited reference

^[11]^.

## Acknowledgments

We thanked the patients who participated in this study and gave permission for us to report details. We thank Angela Jansen, Ph.D., MHS of Angela Jansen & Associates, for her editorial services in preparing the manuscript for publication.

## Author contributions

All authors contributed to and approved the final manuscript.

**Clinical data acquisition:** Anh-Hoa Nguyen Pham, Kim-Oanh Bui Thi, and Mai-Huong Nguyen Thi.

**Conceptualization:** Anh-Hoa Pham, Minh-Dien Tran.

**Critical revision of the manuscript for intellectual content:** Anh-Hoa Nguyen Pham, Diem-Ngoc Ngo, Nakayuki Naritaka, Hiroshi Nittono, Hisamitsu Hayashi, Hoa Giang, Hoai-Nghia Nguyen, Hung-Sang Tang, Dinh-Kiet Truong, and Minh-Dien Tran.

**Data curation:** Anh-Hoa Pham, Hoa Giang, Kim- Nguyen, Kim-Oanh Thi, Mai-Huong Thi.

**Drafting the manuscript:** Anh-Hoa Nguyen Pham and Tat-Thanh Nguyen.

**Formal analysis:** Anh-Hoa Pham.

**Funding acquisition:** Anh-Hoa Pham.

**Investigation:** Anh-Hoa Pham, Diem-Ngoc Ngo, Dinh-Kiet Truong, Hoai-Nghia Nguyen, Hung-Sang Tang, Kim- Nguyen, Kim-Oanh Thi, Mai-Huong Thi, Trang Dao.

**Laboratory work:** Trang Thi Dao, Mai-Huong Nguyen Thi, and Hoa Giang.

**Methodology:** Anh-Hoa Pham, Diem-Ngoc Ngo, Hoa Giang, Hoai-Nghia Nguyen, Hung-Sang Tang, Kim-Oanh Thi.

**Obtaining Funding:** Hoai-Nghia Nguyen and Dinh-Kiet Truong.

**Patient care and follow up:** Anh-Hoa Nguyen Pham, Kim-Oanh Bui Thi, Mai-Huong Nguyen Thi, Diem-Ngoc Ngo, Tat-Thanh Nguyen, and Hung-Sang Tang.

**Project administration:** Anh-Hoa Pham, Mai-Huong Thi.

**Resources:** Anh-Hoa Pham.

**Study concept and design:** Anh-Hoa Nguyen Pham, Minh-Dien Tran, Hung-Sang Tang, and Tat-Thanh Nguyen.

**Supervision:** Anh-Hoa Pham, Hiroshi Nittono, Hisamitsu Hayashi, Hoai-Nghia Nguyen, Nakayuki Naritaka.

**Validation:** Anh-Hoa Pham.

**Writing – original draft:** Tat-Thanh Nguyen.

**Writing – review & editing:** Anh-Hoa Pham.
